# Load Transference with the Gain of Excessive Body Mass: A Two-Year Longitudinal Study

**DOI:** 10.3390/ijerph18062879

**Published:** 2021-03-11

**Authors:** Ruoyi Li, Qingyun Liu, Xuecan Chen, Shiyang Yan, Yihong Zhao, Linshan Zhang, Jitka Badurova, Luming Yang, Haojun Fan

**Affiliations:** 1 National Engineering Research Center of Clean Technology in Leather Industry, Sichuan University, Chengdu 610065, China; 2017323080014@stu.scu.edu.cn (R.L.); liuqingyun12@stu.scu.edu.cn (Q.L.); shiyangy@kth.se (S.Y.); zhaoyihong@stu.scu.edu.cn (Y.Z.); zhanglinshan@stu.scu.edu.cn (L.Z.); 2 Key Laboratory of Leather Chemistry and Engineering, Sichuan University, Chengdu 610065, China; fanhaojun@scu.edu.cn; 3 Rongcheng Customs District P.R. China, Fuzhou 350015, China; rchgxxgk@Customs.gov.cn; 4 Faculty of Technology, Tomas Bata University, 76001 Zlin, Czech Republic; jitka.badurova@gmail.com

**Keywords:** foot function, overweight children, obese children, load transfer, follow-up study

## Abstract

Previous studies investigating the effect of excessive weight on the foot have commonly been cross-sectional; therefore, it is still unclear how the foot function gradually changes with the increased body mass that is physiologically gained over time. This study aimed to use a load transfer method to identify the mechanism of how the foot function changed with the increased excessive body mass over two years. Taking normal weight as the baseline, fifteen children became overweight or obese (group 1), and fifteen counterparts maintained normal weight (group 0) over the two years. Barefoot walking was assessed using a Footscan^®^ plate system. A load transfer method was used based upon the relative force–time integral (FTI) to provide an insight into plantar load transference as children increased in weight. Significantly increased FTIs were found at the big toe (BT), medial metatarsal (MM), lateral metatarsal (LM), and lateral heel (HL) in group 1, while at BT, MM, medial heel (HM), and HL in group 0. Foot load showed a posterior to anterior transferal from midfoot (2.5%) and heel (7.0%) to metatarsal and big toe in group 1. The control group, however, shifted the loading within the metatarsal level from LM to HM (4.1%), and equally relieved weight from around the midfoot (MF) (3.0%) to BT, MM, HM and HL. Earlier weight loss intervention is required to prevent further adverse effects on foot functions caused by excessive weight-bearing.

## 1. Introduction

Excessive body mass is known to be strongly associated with the development of musculoskeletal disorders, lower extremity postural deformities, and altered walking characteristics for children [1,2,3,4]. Especially, as the foot structure is in the process of development in children, childhood obesity may undermine the foot structure and foot function and further lead to the redistribution of foot loadings [5,6,7,8]. Previous studies investigating the effect of increased weight on the plantar pressures and foot structural changes in children have commonly been cross-sectional [9,10,11,12]; obese/overweight children are compared with those of normal weight, but the temporal relationship of pathologic development on foot function with the increased excessive body mass is unknown. It is necessary to figure out how the foot function gradually changes with the increased excessive body mass that is physiologically gained over time. Additionally, the foot structure maintains constant growth until the age of 12 or 13 years [13,14]. It is notable that with the increase in excessive body mass, distinctions exist between physiologic and pathologic development in children. Therefore, a longitudinal study is needed to evaluate pathologic development with the gain of excessive body mass.

Load transfer analysis has been used in previous studies [15,16,17,18]. Bus et al. firstly designed a load-transfer algorithm, which was used to assess load redistribution resulting from custom-made insoles [15,16,17]. Mingyu Hu et al. also used a load transfer algorithm to quantify the plantar force transference to observe the track of the load under children’s feet [19]. Likewise, the load transfer analysis method would be a useful tool to figure out the redistribution of foot loadings with the increased body mass in the present study. To date, most studies have focused on the comparison of plantar pressure distribution, but the correlation between the varied parts of the foot structure with load redistribution is not usually considered. The load transfer analysis can provide an insight into the mechanism of load redistribution with increased body mass, and could help to achieve a clear understanding of the temporal relationship of pathologic development with the increased excessive body mass. Accordingly, a load transfer algorithm designed by Mingyu Hu et al. [19] was used in this study.

Therefore, the aim of this study was to identify the load transference with the increased excessive body mass and to establish the mechanism about how foot function changes with the increased excessive body mass over two years.

## 2. Methods

### 2.1. Participants

In total, 158 children aged 7–9 years from a randomly selected local primary school in Yantai City, China, participated in the original study. Participants were excluded if they had any of the following: neurological and orthopedic problems, a history of lower limb injury during the previous 6 months, and previous foot surgery. The original and follow-up measurements were conducted in September 2017, and September 2019, respectively. This study was approved by the Ethics Committee of Sichuan University (K2020044). Written informed consent was obtained from one of the children’s guardians before the experiments. Body mass index (BMI) was calculated as the body mass divided by height squared (kg·m^−2^). Participants were categorized as normal-weighted, overweight, and obese according to the BMI reference norm established by the Group of China Obesity Task Force (Table 1) [20].

### 2.2. Equipment and Procedure

Anthropometric data, including age, gender, height, and body mass, were recorded at baseline and follow-up. Plantar pressure parameters were measured by a one-meter Footscan^®^ plate system (RSscan International, Olen, Belgium) with a sampling frequency of 250 Hz. The platform was positioned at the center of a 10 m walkway. After familiarization, participants were instructed to walk barefoot across the plate at their preferred speed. A two-step initial protocol was used during data collection [21]. A trial was considered valid when the following criteria were met: a natural walk with self-preferred speed and two whole steps of both feet were recorded by the plate system. At least three successful trials were recorded for each participant, and the mean values of the right foot were calculated for analyses [22].

### 2.3. Data Processing and Statistical Analysis

For analyzing load transferences between foot regions, the foot plantar was divided into seven anatomical segments [23]: big toe (BT), second–fifth toes (T2–5), medial metatarsal (MM, consisting of first metatarsal, second metatarsal, and third metatarsal), lateral metatarsal (LM, consisting of third metatarsal and fourth metatarsal), midfoot (MF), medial heel (HM), and lateral heel (HL). The force–time integral (FTI) is the total load in a certain region of the foot which indicates the duration of contact so that the overall load in that region can be fully described; therefore, the FTI was calculated for each region to describe the inter-regional load transfer [19]. To eliminate the effects of different body mass between the baseline and follow-up, force–time integral was normalized to relative FTI (FTIrel). The formula was calculated as follows:

$$\mathrm{FTIrel}\;\left(\%\right)=\frac{\mathrm{FTI}\left(\mathrm{foot}\;\mathrm{region}\right)}{\sum \mathrm{FTI}\left(\mathrm{foot}\;\mathrm{region}\right)}\times 100\%$$

The change of foot structure with the increased body mass was assessed by the arch index (AI). The arch index was defined as the ratio of the midfoot contact area relative to the total contact area, excluding the toes [24].

Statistical analyses were conducted with SPSS 21.0 (IBM, New York, NY, USA). Kolmogorov–Smirnov tests and Q–Q normality plots were used to test the data for normality. Independent sample *t*-tests were used to compare the differences between the original data and follow-up data in anthropometry variables and FTI values for each group. Statistical differences between group 1 and group 0 at baseline and follow-up for anthropometry variables and FTI values were also analyzed with an independent samples *t*-test. Confidence intervals (CI) at 95% were calculated for all the mean differences. A value of *p* < 0.05 was perceived as significant for statistical analyses in this study.

### 2.4. Load Transfer Method

Load transfer with the increased body mass was assessed by a load transfer method proposed by Mingyu Hu [19]. The values of load transferences during the two-year follow-up were quantified by the difference values of FTIrel, which were calculated by the mean of baseline data minus that of the follow-up data. Positive values indicated that the forces of the baseline were higher than those in the follow-up; this was vice versa for negative values. Four levels were defined following the anatomical segments: toes (Level 1, BT and T2–5), metatarsal (Level 2, MM and LM), midfoot (Level 3, MF), and heel (Level 4, HM and HL). In the beginning, foot load was transferred within each level from positive value regions to negative value regions. Load transfer occurred between adjacent anatomical regions first, and then between the further regions. Afterwards, the foot load was transferred between adjacent levels from positive value regions to negative value regions. Finally, load was transferred across levels with the help of longitudinal arches. After the transfer, the altered FTIrel values were shown at the bottom of each foot region.

## 3. Results

### 3.1. Participant Characteristics

Of the original 158 participants, 24 of these participants were excluded: 15 participants were unable to attend the scheduled follow-up measurements, 2 participants were due to missing measurement data, and 7 participants because of pathologic foot developments. Therefore, complete datasets of 134 children were available for observation. All of the children were categorized as normal-weighted, overweight, or obese according to their BMI. Children who had increased their BMI from normal weight to overweight or obese after two years were selected. Fourteen children increased their BMI from normal weight to overweight, and only one child increased the BMI from normal weight to obese. Therefore, these fifteen children with increasing BMI were assigned to the study group (group 1). Non-obese counterparts who maintained a BMI of normal weight over the two years were also selected. To counter a possible bias of body factors, fifteen children whose age, gender, and body height were matched to the individuals of group 1 at baseline and follow-up were selected as the control group (group 0). The participants’ characteristics are shown in Table 2.

After the two-year follow-up check, height, weight, and BMI of children in the two groups increased significantly, and the weight and BMI of group 1 showed a greater increase than group 0. The average weights of group 0 and group 1 participants increased by 7.8 kg and 12.4 kg, respectively. The BMI values of group 0 and group 1 participants increased by 1.7 kg·m^−2^ and 3.4 kg·m^−2^, respectively.

### 3.2. Arch Index

After the two-year follow-up check, the AI of all groups decreased significantly. The AI of group 0 decreased from 0.27 to 0.16. The AI of group 1 decreased from 0.27 to 0.19. Group 1 showed greater AI values at follow-up than those in group 0.

### 3.3. FTI

The FTI values of the two groups are shown in Table 3. As the result shows, FTI values of both groups increased in all foot regions after the follow-up check. Significant increases were displayed at BT, MM and HL regions in both groups. It was noticed that the FTI of group 1 showed a significant increase of 26.8 N·s at the LM region (*p* = 0.001), while the increased value in group 0 was only 6.2 N·s (*p* = 0.379) at that region. Meanwhile, the increased FTI value of group 0 in the HM region was 13.6 N·s (*p* = 0.003), and the increased FTI value of group 1 in that region was only 9.9 N·s (*p* = 0.110). Foot loading distributions showed no significant difference between the two groups at baseline and follow-up.

### 3.4. Load Transfer

**Figure 1:**
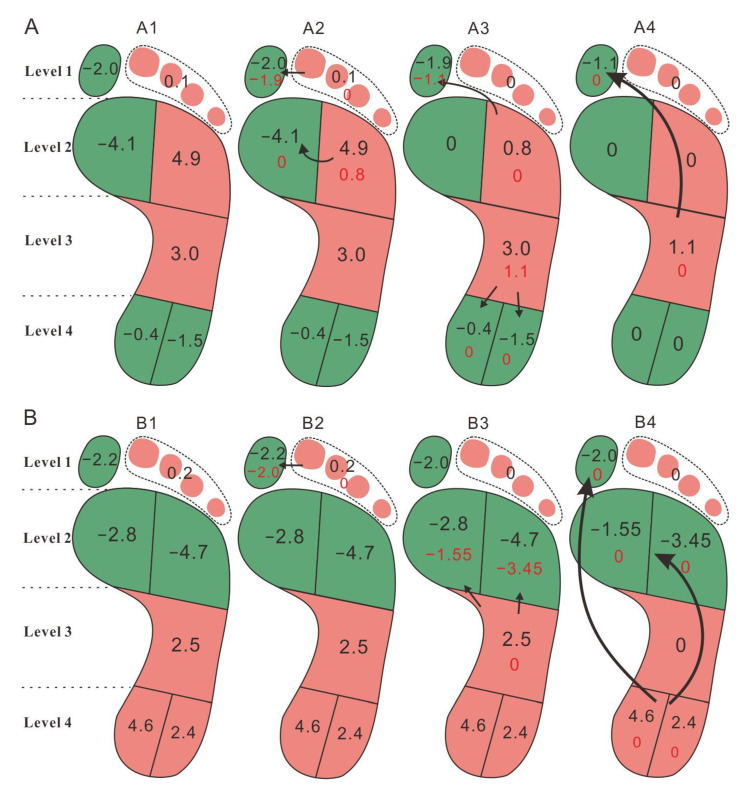
Load transferences in (**A**) group 0 and (**B**) group 1. (**A1**,**B1**) show the transfer values in each foot region. In the beginning, load transfers were within each level (**A2**,**B2**). Arrows going from the positive region (red regions) to the negative region (green regions) means that the loss of foot load in the positive region is transferred to the negative region. Load transference occurs between adjacent anatomical regions first, and then between the further regions. Afterwards, load is transferred between adjacent levels from positive regions to negative regions (**A3**,**B3**). Finally, load transfers were across levels (**A4**,**B4**). The FTIrel values after transference are shown at the bottom of each foot region.

Calculated FTIrel and transfer values of all groups are shown in Table 4. The assessments of load transference in group 0 and group 1 are illustrated in Figure 1. The calculated transfer values were matched to the foot regions (Figure 1A,B).

As shown in Figure 1, with a two-year development in weight-increased children, MF, HM, and HL were the main regions to where the load was transferred in group 1. Foot load showed a posterior to anterior transferal from the midfoot and heel to the metatarsal and big toe. The control group, however, shifted the loading within the metatarsal level from LM to HM, and equally relieved weight from around MF to BT, MM, HM and HL. Notably, foot loading was relieved from MF regions, as well as being concentrated in BT and MM in both groups. However, different load transferences were displayed in LM, HL, and HM regions between the two groups. Concerning the LM region, load was transferred from this region to BT and MM in group 0 with a decrease in FTIrel from 21.6% to 16.7%; however, the load was transferred from the midfoot and heel to the LM regions in group 1, where the FTIrel increased from 16.5% to 21.2%. Concerning the heel region, the FTIrel of HM and HL in group 0 increased by 0.4% and 1.5%, respectively. This increased FTIrel originated from the MF regions. The FTIrel of heel regions decreased in group 1, because the load had transferred from heel regions to metatarsal regions with the two-year development.

## 4. Discussion

In this two-year follow-up study, load transference in children whose BMI increased from normal weight to overweight or obese was identified, and further compared with normal-weighted children. As the results showed, the gain of excessive body mass led to different load transferences compared to normal-weighted children. After a two-year development in children with increasing BMI, foot load showed a posterior to anterior transferal from the midfoot and heel to the metatarsal and big toe. The normal-weighted children, however, shifted the loading within the metatarsal level from LM to HM, and equally relieved the loads from around the MF to BT, MM, HM, and HL.

Foot-loading was concentrated in BT in both groups, showing a development towards an adult-like foot loading pattern with the increased bone intensity of the big toe [25]. As children increased in body weight from normal-weight to overweight or obese, the excessive body mass led to load transference to the metatarsal regions. Specifically, it was noticed that load was transferred from LM to MM in group 0 with the development of the foot, resulting in a rather large foot loading in the MM region (33.2% FTIrel) compared to that in the LM region (16.7% FTIrel). However, foot loading in metatarsal regions tended to be equally distributed in LM and MM regions in group 1 (26.8% FTIrel, 21.2% FTIrel). The equal distribution was also reported in previous studies [1]; this could be an adaption strategy to compensate for additional bodyweight.

Martínez et al. indicated that the foot posture of children changes from a rather flat foot to a neutral foot type with significantly reduced FPI scores, which has a minimal relationship with the BMI from their three-year prospective study [26]. Similar results were found in AI values in the present study. The AI values significantly decreased during the two-year follow-up both in group 0 and group 1. Meanwhile, the midfoot revealed a continuous reduction in foot loading in group 0 and group 1 with the development of the foot. The results suggested a link between midfoot load transference and foot arch raises. The decreased AI values indicated the maturation of the longitudinal foot arch from a rather flat foot to a normal or even higher arch pattern, accompanied by the resolved plantar soft tissue. With the raises of the longitudinal foot arch, the spring mechanics of the foot arch become active [27]. When the foot is encumbered with load during walking, the longitudinal arch collapses to absorb the foot loading and stores strain energy, subsequently recoiling to generate propulsion. Additionally, with the help of the windlass mechanism, foot load in the midfoot is transferred to other regions with the alleviation of the flatness of the longitudinal foot arch. The AI value in group 1 displayed a smaller decrease than that in group 0. This was attributed to the greater medial midfoot pad thickness and the collapse of the medial longitudinal arch, which resulted from the excessive body mass [28,29]. Additionally, the relieved FTI value of MF in group 1 was lower than that in group 0. A cross-sectional study has indicated that the feet of overweight and obese children follow a different growth pattern from that of normal-weight children [30]. The delayed development of the foot and the collapse of the medial foot arch brought by the excessive body mass could lead to a reduced ability of the foot arch to absorb shock and alleviate foot loading than that in normal-weight children.

Force–time integral (FTI) is the total load in a certain region of the foot which indicates the duration of contact; thus, the overall load in that region can be fully described. Numerous studies have indicated that obesity could lead to greater force–time integrals under metatarsal, midfoot and heel regions in comparison to normal-weighted children [31]. In this study, with the gain of excessive body mass, significantly increased FTIs were found under BT, MM and HL regions in both groups. Moreover, the LM region displayed a significantly increased FTI in group 0, and the HM regions displayed a significantly increased FTI in group 1. These differences can be explained by load transference in the two groups. BT and MM were the two main regions to where the load was transferred, both in group 0 and group 1, although different load transferences were displayed in LM and HL between the two groups. Load was transferred from HL to LM in group 1, but transferred from lateral to medial within the metatarsal level, and from MF to HL in group 0. Because load is transferred from midfoot and heel to the big toe and metatarsal with the gain of excessive body mass, the big toe and metatarsal regions are more exposed to high foot loading than normal. Additionally, the force–time integrals were normalized to relative FTI, which can directly reflect the foot loading distribution among all the foot regions. The main loading regions in the two groups were the metatarsal and heel regions. Despite the different load transferences in group 0 and group 1, the foot loading distributions showed no significant difference between the two groups. This may be attributable to the excessive weight-bearing having been maintained for enough time to adversely change the foot function. Therefore, the authors suggest that weight loss interventions should be taken as early as possible.

There were two strengths in our study. Firstly, this was the first study to use a load transfer method to investigate load transference with increased excessive body mass, which provided an insight into the mechanism of load redistribution with weight increases. Secondly, a two-year longitudinal investigation was conducted in this study, making it possible to determine how the foot gradually changed with the increased excessive body mass that was physiologically gained over time. However, two main limitations existed in this study. Firstly, it will be necessary to add a larger sample to the study cohort to reinforce the results. Secondly, a longer duration of follow-up is needed to investigate the variation of load transferences.

## 5. Conclusions

In this study, load transference with the gain of excessive body mass in children whose BMI increased from normal-weight to overweight or obese was identified, and compared with the normal. As the results showed, the gain of excessive body mass led to different load transferences compared to normal-weighted children. Foot load showed a posterior to anterior transferal from the midfoot and heel to the metatarsal and big toe regions with the gain of excessive body mass. The control group, however, shifted the loading within the metatarsal level from LM to HM, and equally relieved load from around MF to BT, MM, HM, and HL. Load was transferred from the midfoot and heel to the big toe and metatarsal with the gain of excessive body mass; therefore, the big toe and metatarsal regions were more exposed to high foot loading than normal. The authors suggest that earlier weight loss intervention is required to prevent further adverse effects on foot functions caused by excessive weight-bearing.

## Figures and Tables

**Table 1:** China BMI reference norms (7–11 years).

Age	Male		Female	
	Overweight	Obese	Overweight	Obese
7	17.4	19.2	17.2	18.9
8	18.1	20.3	18.1	19.9
9	18.9	21.4	19.0	21.0
10	19.6	22.5	20.0	22.1
11	20.3	23.6	21.1	23.3

**Table 2:** Participant characteristics (Mean ± SD).

	Group	Baseline	Follow-Up	Mean Difference (95% CI)	*p*
Number	Group 0	15	15	-	-
	Group 1	15	15	-	-
Age (years)	Group 0	7.7 ± 0.5	9.7 ± 0.5	2	-
	Group 1	7.7 ± 0.5	9.7 ± 0.5	2	-
Height (cm)	Group 0	130.3 ± 4.1	141.2 ± 4.8	11.0 (9.9 to 12.0)	**0.000**
	Group 1	129.9 ± 4.3	141.1 ± 4.7	11.3 (10.3 to 12.2)	**0.000**
Weight (kg)	Group 0	25.4 ± 2.6	33.3 ± 4.9	7.8 (6.5 to 9.2)	**0.000**
	Group 1	**28.0 ± 2.3**	**40.4 ± 3.8**	12.4 (11.0 to 13.8)	**0.000**
BMI (kg·m^−2^)	Group 0	15.0 ± 1.3	16.6 ± 2.0	1.7 (1.2 to 2.2)	**0.011**
	Group 1	**16.6 ± 0.8**	**20.3 ± 0.9**	3.6 (3.1 to 4.2)	**0.000**
AI	Group 0	0.27 ± 0.07	0.16 ± 0.07	−0.11 (−0.08 to −0.13)	**0.000**
	Group 1	0.27 ± 0.06	0.19 ± 0.06	−0.08 (−0.06 to −0.10)	**0.002**

Mean Difference represents the mean difference in anthropometry variables between the baseline and the follow-up. *p*-values represent the differences in anthropometry variables between the baseline and the follow-up. The bold represents statistical differences between the two group at baseline and follow-up.

**Table 3:** FTI (N·s) and the difference values.

Regions	Group 0				Group 1			
	Baseline	Follow up	Mean Difference (95% CI)	*p*	Baseline	Follow up	Mean Difference (95% CI)	*p*
BT	6.7 ± 3.0	16.2 ± 8.9	9.5 (5.0 to 14.0)	**0.001**	6.0 ± 4.6	16.9 ± 10.4	10.9 (6.4 to 15.5)	**0.001**
T2–5	2.2 ± 1.7	3.3 ± 2.1	1.0 (-0.4 to 2.5)	0.163	1.8 ± 1.5	3.4 ± 3.1	1.6 (−0.2 to 3.4)	0.082
MM	27.4 ± 9.2	60.0 ± 22.0	34.4 (21.6 to 47.2)	**0.000**	24.4 ± 15.4	55.2 ± 25.2	30.8 (14.9 to 46.6)	**0.000**
LM	25.2 ± 19.6	31.4 ± 18.0	6.2 (−4.4 to 16.8)	0.379	17.9 ± 10.6	44.7 ± 23.0	26.8 (14.2 to 39.3)	**0.001**
MF	13.7 ± 13.3	15.9 ± 13.6	2.2 (−1.9 to 6.3)	0.663	17.3 ± 15.7	23.4 ± 16.9	7.3 (−3.0 to 17.6)	0.320
HM	17.0 ± 8.7	30.6 ± 13.1	13.6 (4.9 to 22.2)	**0.003**	23.1 ± 18.3	33.1 ± 14.5	9.9 (−1.0 to 20.9)	0.110
HL	14.0 ± 6.9	28.1 ± 13.4	14.0 (6.4 to 21.6)	**0.002**	19.0 ± 11.1	31.0 ± 12.3	12.0 (4.1 to 19.9)	**0.009**

Mean Difference represents the mean difference in FTI values between the baseline and the follow-up. *p*-values represent the differences in FTI values between the baseline and the follow-up. Abbreviations: big toe (BT), second–fifth toes (T2–5), medial metatarsal (MM), lateral metatarsal (LM), midfoot (MF), medial heel (HM), and lateral heel (HL).

**Table 4:** FTIrel (%) and the transfer values.

Regions	Group 0			Group 1		
	Baseline	Follow up	Transfer Value	Baseline	Follow up	Transfer Value
BT	6.6	8.6	−2.0	6.2	8.4	−2.2
T2–5	1.9	1.8	0.1	1.9	1.7	0.2
MM	29.1	33.2	−4.1	24.0	26.8	−2.8
LM	21.6	16.7	4.9	16.5	21.2	−4.7
MF	11.0	8.0	3.0	13.3	10.8	2.5
HM	16.1	16.5	−0.4	20.4	15.8	4.6
HL	13.6	15.1	−1.5	17.6	15.2	2.4

The transfer value is calculated as the baseline FTIrel value minus the follow-up FTIrel value. Abbreviations: big toe (BT), second–fifth toes (T2–5), medial metatarsal (MM), lateral metatarsal (LM), midfoot (MF), medial heel (HM), and lateral heel (HL).

## Data Availability

The data presented in this study are available on request from the corresponding author.

## References

[1] Steinberg N., Nemet D., Pantanowitz M., Eliakim A. (2018). Gait Pattern, Impact to the skeleton and postural balance in overweight and obese children: A review. Sports.

[2] Mignardot J.-B., Olivier I., Promayon E., Nougier V. (2010). Obesity impact on the attentional cost for controlling posture. PLoS ONE.

[3] Anandacoomarasamy A., Caterson I., Sambrook P., Fransen M., March L. (2008). The impact of obesity on the musculoskeletal system. Int. J. Obes..

[4] Gil Madrona P., Romero Martinez S.J., Saez-Gallego N.M., Ordonez Camacho X.G. (2019). Psychomotor limitations of overweight and obese five-year-old children: Influence of body mass indices on motor, perceptual, and social-emotional skills. Int. J. Environ. Res. Public Health.

[5] Mesquita P.R., Neri SG R., Lima R.M., Carpes F.P., de David A.C. (2018). Childhood obesity is associated with altered plantar pressure distribution during running. Gait Posture.

[6] Song-Hua Y., Lu W., Kuan Z. (2017). Effects of different movement modes on plantar pressure distribution patterns in obese and non-obese Chinese children. Gait Posture.

[7] Steele J.R., Riddiford-Harland D.L., Mickle K.J. (2015). Excessive weight bearing compromises foot structure and function across the lifespan. Mechanobiol. Obes. Relat. Dis..

[8] Wyszynska J., Leszczak J., Podgorska-Bednarz J., Czenczek-Lewandowska E., Rachwal M., Deren K., Baran J., Drzal-Grabiec J. (2020). Body fat and muscle mass in association with foot structure in adolescents: A cross-sectional study. Int. J. Environ. Res. Public Health.

[9] Da Rocha E.S., Bratz D.T., Gubert L.C., de David A., Carpes F.P. (2014). Obese children experience higher plantar pressure and lower foot sensitivity than non-obese. Clin. Biomech..

[10] Yan S.H., Zhang K., Tan G.Q., Yang J., Liu Z.C. (2013). Effects of obesity on dynamic plantar pressure distribution in Chinese prepubescent children during walking. Gait Posture.

[11] Gijon-Nogueron G., Montes-Alguacil J., Martinez-Nova A., Alfageme-Garcia P., Cervera-Marin J.A., Morales-Asencio J.M. (2017). Overweight, obesity and foot posture in children: A cross-sectional study. J. Paediatr. Child Health.

[12] Catan L., Amaricai E., Onofrei R.R., Popoiu C.M., Iacob E.R., Stanciulescu C.M., Cerbu S., Horhat D.I., Suciu O. (2020). The impact of overweight and obesity on plantar pressure in children and adolescents: A systematic review. Int. J. Environ. Res. Public Health.

[13] Forriol F., Pascual J. (1990). Footprint analysis between 3 and 17 years of age. Foot Ankle.

[14] Volpon J.B. (1994). Footprint analysis during the growth period. J. Pediatric Orthop..

[15] Bus S.A., Ulbrecht J.S., Cavanagh P.R. (2004). Pressure relief and load redistribution by custom-made insoles in diabetic patients with neuropathy and foot deformity. Clin. Biomech..

[16] Bus S.A., van Deursen R.W., Kanade R.V., Wissink M., Manning E.A., van Baal J.G., Harding K.G. (2009). Plantar pressure relief in the diabetic foot using forefoot offloading shoes. Gait Posture.

[17] Bus S.A., Waaijman R., Arts M., Manning H. (2009). The efficacy of a removable vacuum-cushioned cast replacement system in reducing plantar forefoot pressures in diabetic patients. Clin. Biomech..

[18] Zhao Y., Zheng D., Yan S., Liu M., Yang L. (2020). Children with obesity experience different age-related changes in plantar pressure distributions: A follow-up study in china. Int. J. Environ. Res. Public Health.

[19] Hu M., Zhou N., Xu B., Chen W., Wu J., Zhou J. (2017). The mechanism of force transference in feet of children ages two to six. Gait Posture.

[20] Group of China Obesity Task Force (2004). Body mass index reference norm for screening overweight and obesity in Chinese children and adolescents. Chin. J. Epidemiol..

[21] Meyersrice B., Sugars L., McPoil T., Cornwall M.W. (1994). Comparison of 3 methods for obtaining plantar pressures in nonpathologic subjects. J. Am. Podiatr. Med Assoc..

[22] Menz H.B. (2004). Two feet, or one person? Problems associated with statistical analysis of paired data in foot and ankle medicine. Foot.

[23] Gerych D., Tvrznik A., Prokesova EV A., Nemeckova Z., Jelen K. (2013). Analysis of Peak Pressure, Maximal Force, And Contact Area Changes during Walking and Running with Conventional and Shock-Absorbing Insoles In the Combat Boots Of the Czech Army. J. Mech. Med. Biol..

[24] Cavanagh P.R., Rodgers M.M. (1987). The arch index—A useful measure from footprints. J. Biomech..

[25] Bosch K., Gerss J., Rosenbaum D. (2010). Development of healthy children’s feet--nine-year results of a longitudinal investigation of plantar loading patterns. Gait Posture.

[26] Martínez-Nova A., Gijón-Noguerón G., Alfageme-García P., Montes-Alguacil J., Evans A.M. (2018). Foot posture development in children aged 5 to11 years: A three-year prospective study. Gait Posture.

[27] Ker R.F., Bennett M.B., Bibby S.R., Kester R.C., Alexander R.M. (1987). The spring in the arch of the human foot. Nature.

[28] Riddiford-Harland D.L., Steele J.R., Baur L.A. (2011). Are the feet of obese children fat or flat? Revisiting the debate. Int. J. Obes..

[29] Dowling A.M., Steele J.R., Baur L.A. (2001). Does obesity influence foot structure and plantar pressure patterns in prepubescent children?. Int. J. Obes..

[30] Jimenez-Ormeno E., Aguado X., Delgado-Abellan L., Mecerreyes L., Alegre L.M. (2013). Foot morphology in normal-weight, overweight, and obese schoolchildren. Eur. J. Pediatrics.

[31] Hills A.P., Hennig E.M., Byrne N.M., Steele J.R. (2002). The biomechanics of adiposity—Structural and functional limitations of obesity and implications for movement. Obes. Rev. Off. J. Int. Assoc. Study Obes..

